# Paediatric regional anaesthesia and peripheral nerve blocks in paediatric outpatient surgery. Prospective, single-center, pragmatic comparative cohort study

**DOI:** 10.1007/s00383-026-06606-0

**Published:** 2026-08-26

**Authors:** Dolev Perez, Gaudat Jaber, Binyamin B. Neeman, Galiya Raisin, Avital Zeldin, Yaron Armon, Elena Feldman, Yaacov Gozal, Israel A. Ostrovsky, Boris Chertin

**Affiliations:** 1 Faculty of Medicine, Department of Urology, Shaare Zedek Medical Center, Hebrew University of Jerusalem, P.O. B 3235, Jerusalem, 91031 Israel; 2 Faculty of Medicine, Department of Paediatric Urology, Shaare Zedek Medical Center, Hebrew University of Jerusalem, Jerusalem, Israel; 3 Faculty of Medicine, Department of Paediatric Surgery, Shaare Zedek Medical Center, Hebrew University of Jerusalem, Jerusalem, Israel; 4 Faculty of Medicine, Department of Anaesthesia, Shaare Zedek Medical Center, Hebrew University of Jerusalem, Jerusalem, Israel

**Keywords:** Paediatric regional anaesthesia, Paediatric urologic surgery, Postoperative pain management, Peripheral nerve blocks

## Abstract

**Background:**

Adequate perioperative analgesia is a key component of paediatric urologic surgery. Several regional anaesthesia techniques are commonly used for inguinal and genital procedures; however, prospective comparative data across different block types remain limited.

**Methods:**

This prospective, single-center, pragmatic comparative cohort study included 160 children undergoing outpatient inguinal or genital urologic surgery. Regional anesthesia was provided using caudal block (CB), quadratus lumborum block (QLB), pudendal block (PDB), or penile block (PB) (*n* = 40 per group), allocated in a procedure-driven, non-randomized manner reflecting routine clinical practice and the personal experience already gained. Primary outcomes were intraoperative opioid requirements and postoperative pain scores at 1, 6, and 24 h. Secondary outcomes included time first to rescue analgesic, postoperative analgesic consumption, post-anaesthesia care unit stay, time to return to regular activity, and block-related complications.

**Results:**

For inguinal procedures, CB and QLB demonstrated comparable analgesic effectiveness; CB was associated with lower intraoperative opioid use, while QLB showed a trend toward lower early postoperative pain scores. In genital procedures, PB was associated with higher pain scores, increased rescue analgesic requirements, earlier need for rescue analgesia, and longer recovery time compared with CB and PDB (*p* < 0.001). No block-related complications were observed.

**Conclusion:**

In this prospective pragmatic cohort, all regional anaesthesia techniques demonstrated acceptable short-term safety. Within procedure-specific comparisons, QLB showed a trend toward improved early analgesia in inguinal surgery, while PDB was associated with more consistent early analgesia than PB in genital procedures. Given the non-randomized design, these findings should be considered exploratory and support a procedure-specific approach to regional block selection in paediatric urologic surgery.

## Introduction

Effective perioperative pain management is a cornerstone of paediatric surgical care, as it directly influences recovery outcomes, reduces complications, and enhances patient and parental satisfaction [1−2]. Over the past decades, regional anaesthesia (RA) has gained popularity in paediatric surgery, providing reliable perioperative analgesia, facilitating early mobilization and discharge, and reducing systemic opioid use [3−4].

For infraumbilical and genital surgeries, several regional techniques are commonly used. Caudal block (CB) remains the most widely used approach due to its simplicity and versatility for both genital and groin surgeries [5]. However, the analgesic duration of single-shot caudal anaesthesia with local anesthetics alone is limited, frequently necessitating supplemental analgesics. The addition of adjuvants such as morphine has been shown to prolong the analgesic effect of caudal anesthesia. We have previously demonstrated that the addition of low-dose morphine to caudal anaesthesia reduced rescue requirements, provided longer-lasting analgesia, and shortened recovery time compared with local anesthetic alone [6].

Penile block (PB) and Pudendal nerve block (PDB) are frequently chosen for genital procedures, providing targeted analgesia to the penile and perineal innervation [7, 8]. In a randomized trial, Rotem et al. demonstrated that CB and PB both outperformed systemic opioids. However, PB was associated with higher postoperative analgesic requirements and earlier need for rescue medication [9]. Similarly, trials in hypospadias repair have shown that PDB provides superior, more prolonged analgesia than CB. Notably, some controversy remains regarding the potential effects of caudal block on urethroplasty outcomes [7].

The quadratus lumborum block (QLB) has emerged as a more recent alternative to inguinal procedures. Targeting the fascial plane near the quadratus lumborum muscle provides both somatic and visceral analgesia and may reduce opioid needs compared to a caudal block in abdominal and inguinal surgery [10−11]. Despite their widespread use, prospective randomized studies directly comparing multiple regional techniques in paediatric urologic outpatient surgery remain scarce. Most available studies focus on two techniques at a time or on specific surgical subtypes, limiting generalizability. Therefore, uncertainty persists regarding the optimal block for different paediatric urological procedures [12].

We hypothesized that block selection should be tailored to the specific surgical procedure rather than applied as a one-size-fits-all approach. The present prospective trial aimed to compare caudal block, quadratus lumborum block, pudendal nerve block, and penile block in male paediatric patients undergoing outpatient genital or groin surgery.

## Materials and methods

### Patient population and study design

Following approval from the Institutional Review Board, a single-center, prospective, pragmatic comparative cohort study was conducted between February 2023 and May 2024. The study enrolled 160 male paediatric patients scheduled for elective outpatient urological surgery (circumcision, hypospadias repair, orchiopexy, hydrocelectomy, or inguinal repair). Exclusion criteria were congenital spinal or sacral malformations, bleeding disorders or coagulopathy, allergy to local anesthetics, urgent or emergency surgery, or the need for postoperative hospitalization.

### Group allocation

Children were first stratified according to the type of surgery, classified as either inguinal/groin or genital procedures. Within each stratum, patients were assigned to one of four study groups according to the regional block administered: Group 1—caudal block (CB); Group 2—quadratus lumborum block (QLB); Group 3—pudendal nerve block (PDB); and Group 4—penile block (PB).

Block allocation was pragmatic and procedure-driven rather than randomized, reflecting routine experience gained through institutional practice and anesthesiologist preference. Accordingly, specific regional techniques were systematically applied to predefined surgical indications. The caudal block group included children undergoing both inguinal/groin and genital procedures. The quadratus lumborum block group consisted exclusively of patients undergoing inguinal or groin surgeries, for which this fascial plane block was routinely used to provide combined somatic and visceral analgesia. Children in the pudendal and penile block groups underwent genital procedures, including circumcision and other penile surgeries. This pragmatic allocation strategy was intended to reflect real-world clinical practice; therefore, the study should be interpreted as a prospective comparative cohort rather than a randomized controlled trial.

### Anesthetic technique

All blocks were performed after induction by an experienced anesthesiologist under sterile conditions, as we have already previously reported [6, 9]. Standard intraoperative monitoring was used in all patients in accordance with institutional paediatric anesthesia guidelines.

Caudal block (CB): A 22-gauge short bevel needle was advanced through the sacral hiatus. After negative aspiration, 0.5-1 mL/kg of 0.25% bupivacaine with 1 mg/kg lidocaine was injected.

Quadratus lumborum block (QLB): With the patient supine, an ultrasound probe was placed above the iliac crest to identify the quadratus lumborum block. A 22-gauge block needle was advanced in-plane to the posterior fascial plane. After hydrodissection, 0.5–0.7 mL/kg of 0.25% bupivacaine with 1 mg/kg lidocaine was injected incrementally.

Pudendal block (PDB): In lithotomy position, a 25-gauge (or 21-gauge if > 15 kg) needle was inserted at the 3 and 9 o’clock positions near the anal verge until motor response was elicited. After confirmation, 0.3 mL/kg of 0.25% bupivacaine with 1 mg/kg lidocaine was injected.

Penile block (PB): With the patient supine, a 27-gauge needle was inserted subcutaneously at the 10 and 2 o’clock positions at the penile base. After negative aspiration, 0.15 mL/kg of 0.25% bupivacaine was injected bilaterally.

### Outcome measures

The primary outcomes of the study were intraoperative analgesic requirements, including the need for paracetamol, fentanyl, or morphine, as well as postoperative pain scores assessed 1,6, and 24 h. Pain was evaluated using the FLACC scale for children aged < 3 years and the VAS scale for children older than 3 years.

The secondary outcomes included the time to the first rescue analgesic, the total systemic analgesic consumption (paracetamol and ibuprofen) during the first 24 h, the duration of stay in the post- anaesthesia care unit (PACU), the time to resume regular activity, and the incidence of adverse events or block-related complications.

### Statistical analysis

All analyses were conducted using Prism 9 for macOS (Version 9.5.0, November 8, 2022). Continuous variables were tested for normality using the Shapiro-Wilk test and compared using ANOVA or the Kruskal-Wallis test as appropriate. Categorical variables were analyzed with chi-square or Fisher’s exact test. Post-hoc pairwise comparisons were performed using the Mann-Whitney *U* test with Bonferroni correction. Results are reported as mean ± standard deviation (SD) or * n*(%), as appropriate. A *p*-value < 0.05 was considered statistically significant.

## Results

### Baseline characteristics

Baseline characteristics differed across the procedure-driven groups, particularly with respect to age and weight; children in the penile block (PB) group were older and heavier than those in the caudal block (CB), quadratus lumborum block (QLB), and pudendal block (PDB) groups (Table 1). ASA physical status and block induction times were comparable across groups, whereas operative duration varied, with the longest procedures observed in the PDB group (Table 1).

**Table 1:** Baseline characteristics and procedural details in four regional anaesthesia groups

	Group I (CB)	Group II (QLB)	Group III (PDB)	Group IV (PB)	*P*-value
Age (± SD)	1.8 (± 2.1)	4.7 (± 2.1)	2.5 (± 3)	14.7 (± 6.5)	0.000
Weight	11 (± 3.5)	20.6 (± 14.2)	19.3 (± 13.9)	64.2 (± 32.4)	***0.000***
*ASA *(%)					0.686
Class I	37 (92.5%)	36 (90%)	37 (92.5%)	35 (87.5%)	
Class II	3 (7.5%)	3 (7.5%)	3 (7.5%)	5 (12.5%)	
Class III	0	1 (2.5%)	0	0	
Class IV	0	0	0	0	
*Type of operation*					
Orchidopexy	15 (37.5%)	15 (37.5%)	0	0	
Hydrocelectomy	7 (17.5%)	15 (37.5%)	3 (7.5%)	0	
Inguinal hernia	0	10 (25%)	1 (2.5%)	0	
Circumcision	10 (25%)	0	13 (32.5%)	40 (100%)	
Hypospadias	8 (20%)	0	23 (57.5%)	0	
Mean time of block induction	18 (± 8.2)	18 (± 7)	19.1 (± 6.9)	15.7 (± 6.8)	0.055
Mean operation time	21.5 (± 7.0)	25.9 (± 9.7)	29.1 (± 13.1)	21.3 (± 6.7)	***0.0*** ***32***

*Statistical tests* Normality was assessed using the Shapiro–Wilk test. Age, weight, block-induction time, and operative time were compared using the Kruskal–Wallis test. ASA class distributions were compared using Pearson’s chi-square test. Data are presented as mean ± SD or *n*

Bold italic p-values indicate statistical significance (p < 0.05).

The distribution of surgical procedures reflected the procedure-driven block allocation strategy: inguinal procedures predominated in the CB and QLB groups. In contrast, genital procedures were performed in the PDB and PB groups.

## Primary outcomes

### Intraoperative analgesic requirements

Intraoperative analgesic requirements differed by procedure and block type. For inguinal procedures, supplemental fentanyl was required less frequently with CB than with QLB (5% vs. 22.5%, respectively; *p* = 0.041). In genital procedures, PB was associated with substantially greater opioid requirements, with 82.5% of patients receiving fentanyl, compared with the other block groups (both comparisons *p* < 0.001). Detailed opioid-use and dose data are presented in Table 2.

**Table 2:** Primary outcomes and adverse events within 24 h

	Group I (CB)	Group II (QLB)	Group III (PDB)	Group IV (PB)	*P*-value
Pain score, 1 h	2.1 ± 1.0	1.6 ± 0.9	2.2 ± 1.0	3.9 ± 1.0	< 0.001 (PB higher)
Pain score, 6 h	2.0 ± 0.9	1.8 ± 0.8	2.1 ± 0.8	3.1 ± 1.2	***< 0.001*** (PB higher)
Pain score, 24 h	1.6 ± 0.7	1.8 ± 0.9	1.7 ± 0.8	2.4 ± 1.1	***0.022*** (PB higher)
Time to first rescue (min)	182 ± 76	180 ± 62	298 ± 81	156 ± 64	***< 0.001*** (PB shorter)
Number of rescue doses, 24 h	1.4 ± 0.7	1.6 ± 0.8	1.5 ± 0.6	2.8 ± 1.1	***< 0.001*** (PB higher)
Intraoperative opioid use	2/40 (5%), 0.3 ± 0.8 µg/kg	9/40 (22.5%), 0.9 ± 1.5 µg/kg	5/40 (12.5%), 0.6 ± 1.2 µg/kg	33/40 (82.5%), 1.8 ± 1.4 µg/kg	***< 0.001***
PACU stay (min)	92 ± 56.4	120 ± 38.7	116 ± 47	105 ± 48.3	**0.043**
Return to normal activity (d)	3.1± 1.2	2.3 ± 1.0	3.4 ± 1.0	5.2 ± 1.7	***< 0.0001*** (PB slower)
Adverse events (*n*, %)	0	0	0	0	–

*Statistical tests*: Continuous and ordinal outcomes were compared using the Kruskal–Wallis test, followed, when significant, by pairwise Mann–Whitney *U* tests with Bonferroni correction. Intraoperative opioid-use proportions were compared using Pearson’s chi-square test. Adverse events were summarized descriptively. Values are presented as mean ± SD or *n* (%)

Bold italic p-values indicate statistical significance (p < 0.05).

### Postoperative pain and analgesic use

**Fig. 1:**
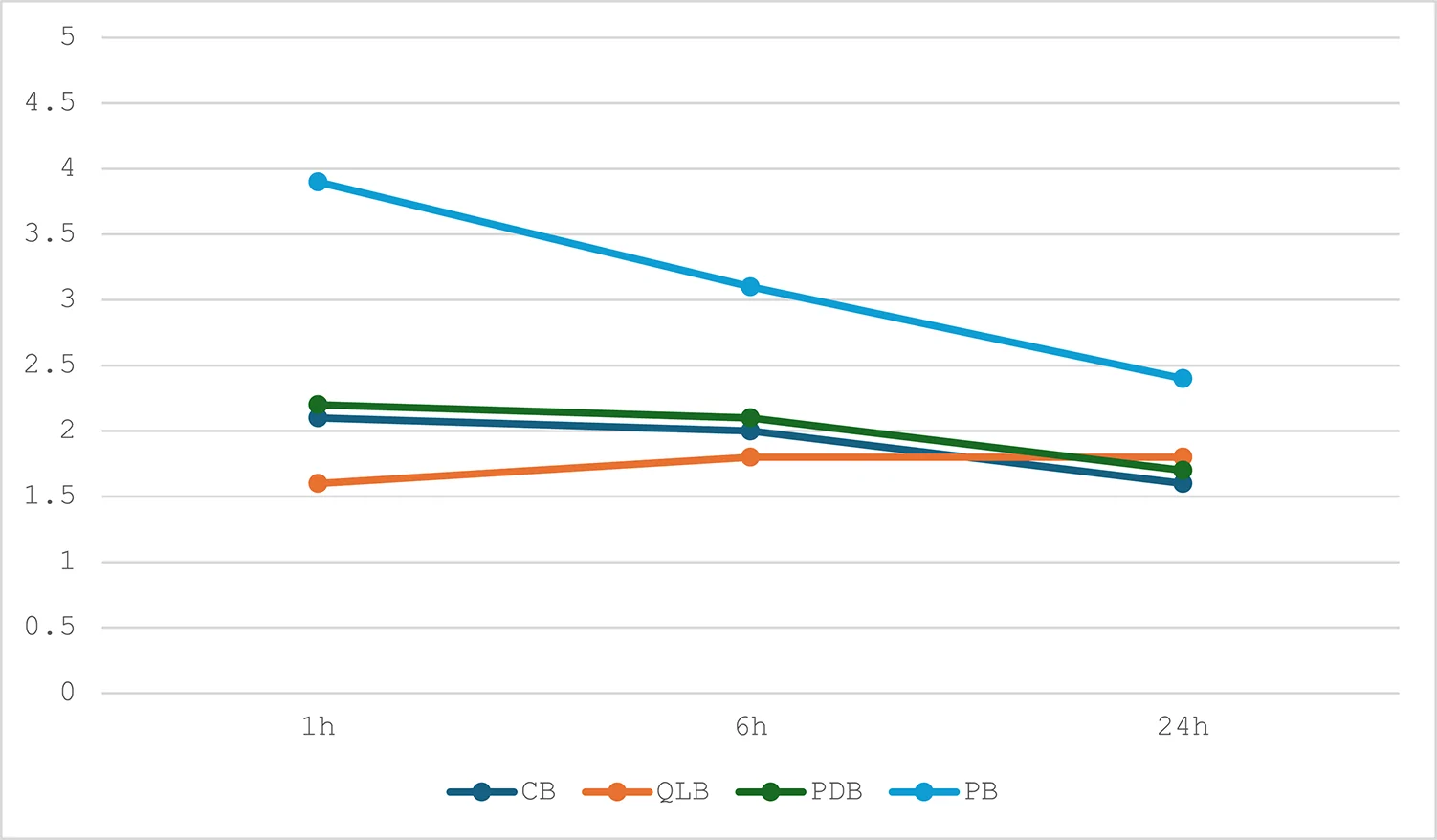
Postoperative pain trajectories (VAS/FLACC) at 1, 6, and 24 h following caudal, quadratus lumborum, pudendal, and penile blocks

At 1 h postoperatively, QLB had the lowest mean pain score (1.6 ± 0.9), whereas PB had the highest (3.9 ± 1.0; *p* < 0.001). PB remained associated with the highest pain scores at 6 and 24 h, although the between-group differences narrowed over time (*p* = 0.022 at 24 h). Pain was assessed using the age-appropriate validated FLACC or VAS scale. Detailed pain scores are presented in Table 2, and their trends are illustrated in Fig. 1.

### Time to first rescue analgesic

Time to first rescue analgesia was similar in the CB and QLB groups but differed across the four block groups overall (*p* < 0.001). The longest interval was observed in the PDB group (298 ± 81 min), whereas the shortest was observed in the PB group (156 ± 64 min). The PB group also required the highest number of rescue doses within 24 h (2.8 ± 1.1; *p* < 0.001). Detailed group data are presented in Table 2.

### Secondary outcomes

#### Time to resume normal activity

Time to return to normal activity differed significantly across the four block groups, with the longest recovery observed in the PB group (5.2 ± 1.7 days; *p* < 0.0001). Detailed group data are presented in Table 2. Normal activity was defined as return to age-appropriate daily activities as reported by caregivers.

### PACU stay

PACU stay differed significantly across the four block groups (*p* = 0.043). The longest mean stay was observed in the QLB group (120 ± 38.7 min), whereas the shortest was observed in the CB group (92 ± 56.4 min). Detailed group data are presented in Table 2.

### Complications

No complications were reported in any group, and all patients were graded 0 on the Clavien-Dindo scale. No early block-related complications were observed, including bleeding, infection, nerve injury, urinary retention, or delayed discharge.

## Discussion

This study prospectively evaluates procedure-specific analgesic profiles of four regional anaesthesia techniques: caudal block (CB), quadratus lumborum block (QLB), pudendal block (PDB), and penile block (PB), used in paediatric outpatient urologic surgeries, categorized by anatomical indication (inguinal vs. genital surgery).

In inguinal procedures, CB and QLB demonstrated comparable overall effectiveness, with CB providing better intraoperative opioid sparing and QLB showing a non-significant trend toward lower early postoperative pain. For genital surgeries, PDB delivered analgesia comparable to CB, whereas PB was less effective, resulting in higher analgesic use, earlier need for rescue medication, and delayed recovery. These findings should be interpreted within procedure-specific contexts rather than as universal superiority of one block over another. These findings generally align with emerging literature. Oksuz et al. [10] reported that QLB yields significantly lower postoperative FLACC pain scores at 4, 6, and 12 h compared to CB in children undergoing inguinal hernia repair and/or orchiopexy, with fewer patients requiring rescue analgesia (*p* < 0.01). A recent systematic review and meta-analysis also demonstrated that QLB reduced the relative risk of requiring rescue analgesia within 24 h compared with CB (RR = 0.37; 95% CI 0.26–0.51; *p* < 0.01) and yielded lower pain scores at 4 and 24 h postoperatively [11].

Our findings are also consistent with those of Rotem et al., who compared ultrasound-guided QLB with CB in paediatric urological surgery. In their mixed prospective–retrospective design (*n* = 41 QLB vs. matched CB controls), postoperative pain scores and time to first rescue analgesia were similar between groups, although block induction time was significantly longer for QLB. Interestingly, the QLB group required somewhat more rescue analgesia within 24 h, while overall satisfaction and complication rates were comparable [9]. Taken together, these data suggest that while QLB represents a safe alternative to CB, particularly when caudal anaesthesia is contraindicated or declined, its routine superiority over CB remains uncertain and warrants confirmation in adequately powered randomized trials.

In genital surgery, PDB appears to offer a favorable analgesic profile. A randomized trial comparing pudendal versus caudal block in hypospadias repair demonstrated significantly lower postoperative pain scores at 12, 18, and 24 h, as well as a prolonged time to first analgesic in the pudendal group (*p* < 0.0001) [13]. In contrast, PB, though easier to administer, was associated in our study with suboptimal analgesia, higher opioid requirements, and slower functional recovery, suggesting that it may be less suitable for more extensive genital procedures. Furthermore, some studies have suggested an association between caudal anaesthesia and increased rates of urethral fistula following hypospadias repair [14]. Although these observations remain controversial and were not evaluated in the present study, they highlight the ongoing interest in alternative regional techniques for genital surgery. In this context, the observed effectiveness of PDB supports its consideration as a viable option in selected paediatric patients. Our findings offer practical insights for anesthesiologists working in paediatric urology. For inguinal procedures, both CB and QLB appear to be reasonable options; CB provides effective intraoperative opioid sparing, while QLB may offer improved early postoperative pain control. In genital surgeries, PDB may achieve a more favorable balance between analgesia and recovery compared with PB. However, given the pragmatic, non-randomized design of this study, these observations should be viewed as exploratory rather than definitive.

All techniques demonstrated acceptable short-term safety, with no block-related adverse events observed. This overall safety profile aligns with existing literature supporting ultrasound-guided regional anaesthesia as a dependable approach with low complication rates [12, 15]. Our study’s strengths include its prospective design, pragmatic real-world setting, inclusion of both inguinal and genital procedures, and direct comparison of four commonly used regional anaesthesia techniques within procedure-specific contexts. This approach enabled the identification of differential analgesic patterns relevant to routine clinical practice. Additionally, the consistent use of validated pain scales and standardized rescue analgesic protocols across groups supports the internal consistency of the collected outcomes.

Our study has several limitations. First, the non-randomized, procedure-driven allocation introduces selection bias and baseline imbalances, particularly with respect to age and body weight, which were not adjusted for analytically. Pain assessment in young children remains inherently subjective, even with validated tools, and inter-rater reliability was not formally assessed. The absence of blinding may have further introduced performance and detection bias, particularly for subjective outcomes. Finally, this single-center study with a modest sample size limits generalizability. However, our findings provide a scientific basis for the future design of a larger randomized study to evaluate different anesthetic techniques in a homogeneous population with the same pathology undergoing the same surgical intervention.

## Conclusions

In this prospective pragmatic cohort study, all four regional anesthesia techniques demonstrated acceptable short-term safety in paediatric outpatient urologic surgery. Within procedure-specific comparisons, the quadratus lumborum block showed a trend toward improved early postoperative analgesia in inguinal procedures, while the pudendal block was associated with more consistent early analgesia than the penile block in genital surgery. These exploratory findings support a procedure-specific approach to regional block selection and warrant confirmation in larger randomized multicenter studies.

## Data Availability

No datasets were generated or analysed during the current study.
